# Popular Trend of Electronic Cigarettes and Their Adverse Effects on Oral Health

**DOI:** 10.7759/cureus.50808

**Published:** 2023-12-19

**Authors:** Mahnoor Fatima, Fatima Muhammad Ali, Rizwan Ullah

**Affiliations:** 1 Dentistry, Jinnah Sindh Medical University, Karachi, PAK

**Keywords:** e-cigarette smoking, oral health care, public health, dental health, electronic cigarette

## Abstract

Electronic cigarettes were originally promoted as a possible tool to assist individuals in quitting smoking, particularly for those who had been tobacco users for an extended period. Compared with traditional tobacco use, these devices were promoted as a safer option. Over the years, it has been proven that conventional cigarettes adversely affect almost all body systems. Owing to the constantly evolving nature of the products and the difficulties in identifying potential e-cigarette effects in traditional tobacco users including combustible and noncombustible forms, studying the impact of e-cigarette usage on oral health is challenging. Although the existing scientific evidence is limited, it indicates that e-cigarette use may have negative effects on oral health. Moreover, the adoption of vaping among young people has increased globally. There is still a lack of awareness regarding the use of e-cigarettes and their associated health complications, especially in developing countries. We aim to sensitize the readers to the pertinent issue, which has clinical and public health significance.

## Editorial

Electronic Nicotine Delivery Systems consist of various devices structurally engineered to produce vapors of a nicotine-containing liquid, with the help of a heating element, which is then inhaled or "vaped." Such devices are commonly referred to as vapes or e-cigarettes. These devices are usually installed with a power source (typically a lithium battery), reservoir containing a liquid, heating element, and mouthpiece for inhalation. Inhaling turns on the battery-powered heating element in e-cigarettes, which turns the liquid into an aerosol to be inhaled [1].

E-cigarettes were primarily introduced and marketed as smoking cessation aids for individuals with a history of chronic smoking, offering reduced exposure to chemicals compared to traditional tobacco consumption. Hon Lik, a Chinese pharmacist, patented the first commercially successful e-cigarette in 2003, intending to provide smokers with a combustion-free alternative to typical tobacco use by delivering nicotine through inhalation [2].

The vaping devices are well known for their flavored vapors due to the presence of propylene glycol, vegetable glycerin, and nicotine in their e-liquid, its prevalence has surged, particularly among adolescents and young adults at a remarkable pace [1]. The prevailing trend of vaping among young individuals is not limited to Western countries but has also impacted Middle Eastern countries as well. In developed countries, the percentage of people who used e-cigarettes between 2009 and 2013 was 7% in Australia, 6% in the United States, and 4% in the United Kingdom. In Asia, Malaysia has the greatest e-cigarette prevalence (14%), followed by the Republic of Korea (7%), and China (0.05%). In 2018, the frequency of e-cigarette consumption was 7.1% in Pakistan, 0.02% in India, and 0.4% in Bangladesh [3].

The oral cavity is the part of the body that is first exposed to e-cigarette aerosols. Therefore, it is susceptible to impairment by any of its components. Oral complications such as ulcers, dry mouth, and inflamed gums were observed in young adults who either vaped, used conventional cigarettes, or used both, as compared to those who refrained from using these substances. Neutrophils, macrophages, and airway epithelium become less resistant after exposure to e-cigarettes, which impairs phagocytosis and reduces epithelial barrier function [4].

The tooth decay associated with e-cigarettes can be caused by several factors. The chemical components of aerosols, including propionaldehyde, lactic acid, and acetic acid, can demineralize enamel and cause changes at the biochemical, cellular, and molecular levels. The presence of a variety of toxins and carcinogens can impact the oral health of smokers by causing inflammation, cytotoxicity, genotoxicity, and decreasing microbial activity, all of which contribute to periodontal diseases. Additionally, artificial flavors in e-cigarettes can make *Streptococcus mutans* adhere to the surfaces of teeth. Recent research has demonstrated that vaping might cause xerostomia, a disorder that promotes the formation of dental caries [5].

E-cigarette users have the goal of quitting smoking and consider it a safer alternative to traditional cigarettes. This is because e-cigarettes are considered less addictive than conventional cigarettes, making them a potential aid for those battling nicotine addiction. However, especially among adolescents, cases of e-cigarette dependence and withdrawal symptoms have been reported. Similarly, the inhalation of metal particles from e-cigarettes, such as chromium, nickel, and lead, has been linked to several negative health impacts such as oxidative stress, reduced cell proliferation, and increased DNA damage. Owing to the adverse effects on oral health associated with e-cigarettes, modern devices have larger batteries for higher-temperature liquid heating. Their lack of a standardized design may pose a risk of oral and facial injuries in the event of device malfunction [1].

In conclusion, e-cigarette use is common in young adults because of the availability of various flavors, discreteness of use, easy accessibility, perceived safety compared to conventional smoking, and targeted advertising and marketing strategies tailored specifically for youth. Control and prevention of the increasing popularity of e-cigarette use, particularly among teenagers, can be achieved through the dissemination of scientific knowledge to teenagers, parents, administrative authorities, and substantial governmental support. E-cigarettes may have some dangerous components, and because there are many distinct types of e-cigarettes and different electronic cigarettes that emit varying levels of harmful chemicals, performing an overall risk evaluation appears to be difficult. Therefore, extensive research needs to be conducted on this subject before labeling it as a "safer alternative."
